# Enrichment of dementia caregiving relationships through psychosocial interventions: A scoping review

**DOI:** 10.3389/fmed.2022.1069846

**Published:** 2023-01-05

**Authors:** Viktoria Hoel, Wei Qi Koh, Duygu Sezgin

**Affiliations:** ^1^Department of Nursing Science Research, Institute for Public Health and Nursing Research, University of Bremen, Bremen, Germany; ^2^School of Nursing and Midwifery, College of Medicine, Nursing and Health Sciences, University of Galway, Galway, Ireland

**Keywords:** dementia, dyadic relationships, caregiving, enrichment, psychosocial interventions

## Abstract

**Objectives:**

Enrichment, defined as “the process of endowing caregiving with meaning or pleasure for both the caregiver and care recipient” can support relationships between people living with dementia (PLWD) and their caregivers. This study aims to explore (1) the types of psychosocial interventions that may enrich relationships between dementia caregiving dyads, and (2) the components within these psychosocial interventions that may contribute to enrichment.

**Methods:**

A scoping review was conducted based on the Joanna Briggs Institute framework. We operationalized and contextualized core elements from Cartwright and colleagues’ enrichment model, which was also used to guide the review. Five electronic databases were searched. Psychosocial intervention components contributing to enrichment were identified and grouped within each core element.

**Results:**

Thirty-four studies were included. Psychosocial interventions generating enrichment among dyads mainly involved supporting dyadic engagement in shared activities, carer education or training, or structural change to the environment around PLWD. Intervention components contributing to the enrichment of dyadic relationships were identified within “acquired symbolic meaning”, “performing activity”, and “fine tuning”. Dyadic communication support and skill-building were common contributors to enrichment.

**Conclusion:**

Our findings may inform the planning and development of interventions to enrich dyadic relationships in the context of dementia. In formal caregiving contexts, future interventions may consider dedicating space for relationships to build and grow through positive interactions. In informal caregiving contexts, existing relationships should be considered to better support dyads engage in positive interactions.

## 1. Background

As described by Kitwood (1, 2), person-centered care has been adopted as the main principle of dementia care. A central theme within the person-centered care model entails safeguarding the “personhood” of individuals with dementia, defined as “a standing or status bestowed upon human beings by others in the context of relationship and social being” (2) (p. 8). Although fundamental elements of personhood include the capacity to form and hold relationships, current person-centered interventions for people living with dementia (PLWD) have been criticized for being implicitly individualistic (3, 4) and insufficiently including the caregivers of PLWD (4–6). By considering the needs of each member of the caregiving dyad [defined as “a caregiving relationship consisting of a caregiver and a care recipient” (7)] separately (8), few existing interventions attempt to “enrich” their caregiving relationship through positive shared experiences (9). Around the beginning of this century, Snyder (10) and Lawrence (11) argued that caregiving relationships in dementia were largely overlooked, where few studies explored the dynamics between the dyad members. Since then, *relationship*-centered approaches have gained increasing recognition in caregiving by adequately including the relational dynamic between the care recipient and caregiver (12, 13).

As dementia progresses, the cognitive and functional changes in PLWD often lead to changing relationship dynamics with their caregivers (14), regardless of whether the caregiver is formal (i.e., paid) or informal (i.e., unpaid). Informal caregivers (e.g., family members or friends) might feel they are caring for someone other than the person they once knew (14). Although informal caregivers face challenges, losses, and negative experiences when caring for a loved one with dementia, these impacts may be lessened by continuing positives within the relationship (15–17). Furthermore, psychosocial interventions that support relationship sustenance can help the caregiving dyad to adapt and live well with the condition (18, 19). Also, in a formal caregiving context, such as long-term care (LTC) facilities (e.g., nursing homes), the common symptoms and dementia trajectory influence the conventional relationship between the PLWD and the formal caregiver (e.g., care staff). Despite dynamic healthcare environments, including staff rotations and turnover, the nature of the caregiving activities and residents’ length of stay constitute a unique setting for building relationships between caregivers and care recipients. This, in turn, directly impacts the experience of both PLWD and caregivers (20). Meaningful nurse-resident relationships in LTC have been associated with staff retention (20–22), highlighting the value of relationship-centered care in providing formal staff with a sense of purpose during caregiving. This notion is supported by Killick and Allan, who argue that formal caregivers generally deeply value the connections and relationships they make with their patients (23). Returning to Kitwood’s person-centered care model, personhood has been found to be sustained in relationships where both caregiver and care recipient experience a close emotional bond (24–26).

Regardless of the underlying theoretical concept, facilitating opportunities for PLWD to connect with caregivers is considered an imperative goal for psychosocial interventions (27–29), arguing for increased focus on activities that enrich the caregiving dyad and enhance the positive aspects of the caregiving relationship when coping with dementia. Maintaining social relationships in dementia is also one of the cornerstones in the work of the INTERDEM Social Health Taskforce, a European interdisciplinary collaborative research network focusing on psychosocial interventions in dementia (30–34). In their operationalization of “Social Health”, they postulate this concept as a possible driver for accessing cognitive reserve in PLWD through active facilitation and utilization of social and environmental resources (34). Furthermore, the INTERDEM Social Health Taskforce argues that interventions focusing on improving or maintaining social relationships in dementia can have beneficial effects on social interactions as well as on clinical and social outcomes for PLWD (30, 31). There is a growing body of research measuring relationship quality in caregiving dyads as an outcome of psychosocial interventions. In addition, there is an increased interest in using enriching activities and caregiving focusing on the interactive capabilities of PLWD, which have been shown to provide important ways to enhance social connections (9, 18, 19, 35–37).

However, what constitutes enriching activities? Following the developed definition by Cartwright and colleagues in 1994, we define enrichment in the context of care as “the process of endowing caregiving with meaning or pleasure for both caregiver and care recipient” (29) (p. 32). Nevertheless, the concept of enrichment in dementia caregiving remains unclear. A variety of terminologies has been used to describe different forms of enrichment in dementia caregiving, such as meaningful (20, 38, 39), rewarding (15, 40, 41), connecting (42–44), and positive aspects of caregiving (28, 32, 41, 45, 46). However, little is known about the components of psychosocial interventions that may generate these positive experiences among caregivers and PLWD. Hence, this scoping review focuses on uncovering the fundamentals of enriching components in psychosocial interventions for dementia caregiving dyads rather than the reported outcomes of these interventions. For the purpose of this review, we define psychosocial interventions as “interpersonal interventions concerned with the provision of information, education, or emotional support together with individual psychological interventions addressing a specific health and social care outcome” (47).

### 1.1. Objectives

By identifying which components may generate enrichment in psychosocial interventions for dementia caregiving dyads, this review aims to understand how to support relationships through enriching experiences for both caregiver and care recipient. Two overarching research questions guided this review:

• “What types of psychosocial interventions reported in the literature may enrich relationships between caregiving dyads in a dementia context?”
• “Which components of these psychosocial interventions may contribute to the enrichment of relationships between caregiving dyads?”

To answer our research questions, we built upon and extended the work of Cartwright and colleagues, who developed a model of enrichment in family caregiving for frail older adults (29). Using this model as a point of departure, we extended this definition to include both formal and informal relationships in dementia caregiving. This model was also used to guide the search strategy and data charting process; components of psychosocial interventions that may generate enrichment in dementia caregiving dyads were identified and mapped to the operationalized core enrichment elements (described in detail in the “Methods” section). The three core elements of enrichment have been operationalized following the definition proposed by Cartwright et al. (29), outlined in detail under “Concept” and summarized in Table 1. The process of operationalizing and contextualizing the core elements of enrichment was supported by findings from our preceding empirical studies focusing on dyadic relationships in dementia caregiving (48, 49), as well as the work of the INTERDEM Social Health Taskforce (30–34) (Supplementary Appendix 1). Within this context, this review mapped out existing types of psychosocial interventions and components that contribute to enrichment in dyadic relationships in formal and informal dementia caregiving contexts.

**TABLE 1:** Operationalization of enrichment in dementia and focus of psychosocial interventions contributing to enrichment.

Core elements: definition	Operationalization	Focus of interventions to generate enrichment
**Acquiring symbolic meaning** the significance, value or intent of an activity or an object, with the symbolic property reflecting meaning that transcends the utility of the given object or activity	Shared activities that facilitate positive relationship gains, such as togetherness/closeness (e.g., doing something together or supporting each other) or connectedness (e.g., opportunities to learn more about the life story of the person with dementia, their interests or their capabilities)	(a) Facilitate social interaction between the dyad members**** (b) Support *dyadic* communication**** (c) Enhanced positive experiences in caregiving**** (d) Maintenance of positive and meaningful social relationships
**Performing activity** The observable behaviors in the caregiving situation. For the purpose of this study, performing activity refers to Psychosocial interventions	Interpersonal interventions concerned with the provision of information, education, or emotional support together with individual psychological interventions addressing a specific health and social care outcome	Psychosocial interventions aiming to support*:* (a) Shared activities for the caregiving dyad to engage in together/activities for the PLWD facilitated by carer**** (b) Activity-based therapies involving both dyad members**** (c) Carer education/training (in interaction with care recipient)**** (d) Support groups for both dyad members/focusing on the caregiving relationship
**Fine tuning** Efforts to accommodate the disease trajectory and personal histories in creating enrichment	The influence of social and environmental resources in finding a balance between capacities and limitations. This refers to interventions that are intended to support caregivers to adapt an activity or environment, to support the care recipient to participate in valued activities	(a) Building assets, focusing on potential, and overcoming the consequences of the disease on personal wellbeing**** (b) Supporting the dyad members to adapt and cope with changing abilities and limitations**** (c) Strengthen people with dementia in their forces and capabilities

## 2. Methods

Scoping reviews are valuable where evidence is widely dispersed or emerging and not yet amenable to questions of effectiveness (50). Furthermore, scoping review research questions may draw upon data from any type of evidence and research methodology (51). The purpose of this review is to scope and present an overview of psychosocial interventions promoting enrichment in dyadic relationships in dementia caregiving; therefore, a scoping review methodology (52) was deemed appropriate. This review was informed by the Joanna Briggs Institute’s (JBI) approach to conducting and reporting scoping reviews (51) and utilized the Preferred Reporting Items for Systematic Reviews and Meta-analysis Extension for Scoping Reviews (PRISMA-ScR) (53) to guide the development, conduct and reporting of this review (Supplementary Appendix 2).

### 2.1. Data sources and search strategy

The concept of enrichment in caregiving, especially in dementia, has not been systematically applied in psychosocial interventions aiming to generate positive experiences in caregiving and improve caregiving relationships. Therefore, the developed search strategy was informed by the three core elements of enrichment proposed by Cartwright et al. (29), operationalized and contextualized to caregiving relationships in dementia.

A prior review of relevant literature was conducted using PubMed (MEDLINE) to pilot the initial search terms, and relevant articles were reviewed to identify additional keywords for inclusion. The initial list of search terms was further refined in consultation with an expert research librarian to optimize the specificity and sensitivity of the search strategy. The search strategy was subsequently applied to five electronic databases in March 2022: MEDLINE via Ovid, CINAHL, AgeLine, Cochrane Library, and PsycINFO via Ovid. Key search terms included but were not limited to: “dementia”, “dyad”, “couple”, “family”, “carer”, “nurse”, “staff”, “social interaction” communication”, “social participation”, “intervention”, “psychosocial”, “reminiscence”, “relationship”, “meaningful”, “community”, “neighborhood”, “home dwelling”, “long-term care”, “assisted living facilities”. A complete strategy for MEDLINE is applied available in the (Supplementary Appendix 3).

### 2.2. Study selection

During the study selection, all records were imported into Endnote and deduplicated before going through a two-phase screening process. Phase one included the screening of titles and abstracts using Rayyan, a web application for systematic reviews (54). The first and second authors (VH and WQK) independently screened titles and abstracts, discussed, and resolved discrepancies. Next, VH and WQK independently screened the full texts of included articles, discussed and resolved any conflicts in consultation with the third author (DS) consulted as necessary. The inclusion criteria are shown in the population, concept, and context (PCC) mnemonic in Table 2.

**TABLE 2:** Population, concept, context (PPC) mnemonic.

	Inclusion criteria
**Population**	**Dyads**, comprising of: - Care recipient: people living with dementia, memory problems or mild cognitive impairment AND - Caregiver: formal or informal
**Concept**	- **Psychosocial interventions that facilitate enrichment** in the dyads (i.e., interventions have to include both the care recipient and caregiver. This means that the interventions should include any of the three core elements of enrichment) 1. Shared activities between the care recipient and caregiver that are intended to facilitate positive relationship gains, OR 2. Interventions evolving around the interaction between the caregiver and care recipient to improve psychological and/or social functioning, including wellbeing and cognition, interpersonal relationships and everyday functional abilities, such as activities and daily living skills, OR 3. Interventions that are intended support caregivers to adapt and activity or environment, to support the care recipient to participate in valued activities
**Context**	**No limits applied**, and can include: - Informal caregiving contexts (e.g., community-dwelling dyads) or - Formal caregiving contexts (e.g., day care, residential facilities)

#### 2.2.1. Population

The study population included caregiving dyads, with caregivers being both formal and informal. The target groups of the psychosocial interventions are people living with dementia (regardless of type or severity of the disease), mild cognitive impairment (MCI), or memory problems as the primary diagnosis and their formal or informal caregiver in a dyadic relationship. Specifically, the reported interventions needed to target dyads as a unit, including care recipient and caregiver, or the relationship between the two.

#### 2.2.2. Concept

The variety of terminologes encompassed in “enrichment” disposed the review to subjectivity. To ensure consistent inclusion of relevant studies, the three core elements in the concept of enrichment (29) were operationalized considering terms that broadly fall under the same definition. According to Cartwright and colleagues, the enrichment process represents the integration of three core elements: “acquired symbolic meaning”, “performing activity,” and “fine tuning” (29). The operationalization of these three elements (outlined in Table 1) guided the search strategy and inclusion criteria:

The first core element, *acquired symbolic meaning*, refers to the significance, value or intent of an activity or an object, with symbolic meaning extending beyond the utility of the given object or activity (29). Supported by empirical findings (49), this core element of enrichment is operationalized as *shared activities* that can lay the groundwork for positive relationship gains. A key element of psychosocial interventions is that they serve as a communication channel for PLWD to engage, interact and talk with others (31, 46). Thus, interventions intended to support social interaction and/or communication are also included here.

The second core element, *performing activity*, is described by Cartwright et al. as the observable behaviors in the caregiving situation (29). To extend the model to a dementia caregiving context, *performing activity* encompass psychosocial interventions, as defined above. Operationalized within a dementia caregiving context, this includes interpersonal relationships, wellbeing, cognition, and functioning in everyday activities (33). A central aspect of psychosocial interventions for PLWD involves supporting *social participation* (30, 31), as the quality of participation in social activities (experienced as meaningful to PLWD) can be considered indicative of social relationships and how a person with dementia stays connected with the social environment (31, 55). As such, social participation was also included in this core element.

The final core element, *fine tuning*, involves efforts to accommodate the “frailty” trajectories – as described by Cartwright and colleagues – and histories in creating enrichment (29). In a dementia caregiving context, this includes different activities and environmental modifications according to the capabilities and personal preferences of PLWD. This is in line with the INTERDEM Social Health Taskforce’s recommendations to focus on the remaining capabilities and strengths of PLWD rather than their deficits and cognitive deterioration (31). Hence, *fine tuning* was operationalized as “Interventions intended to support caregivers to adapt an activity or environment, to support care recipient’s participation in valued activities.”

#### 2.2.3. Context

All caregiving contexts were included, as the focus of this review was to assess interventions facilitating enrichment in dyadic relationships, regardless of context.

**FIGURE 1:**
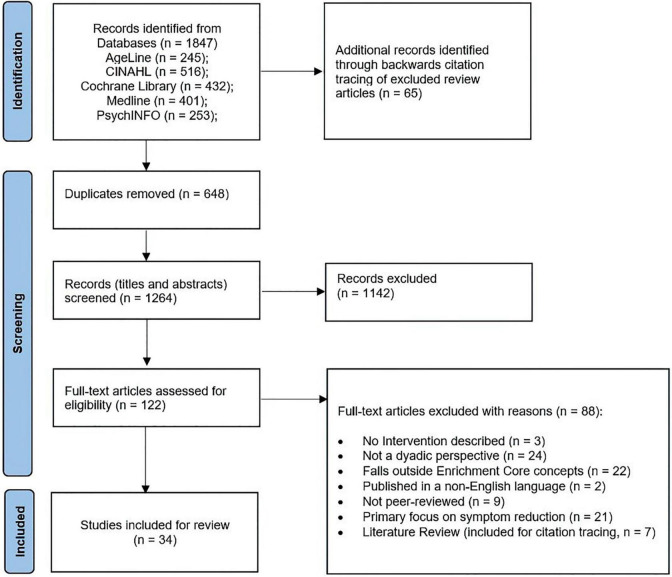
PRISMA flow diagram.

In addition to the outlined PCC, the following inclusion criteria were applied: (i) published, peer-reviewed papers; (ii) qualitative, quantitative or mixed-methods primary research; (iii) reporting on an intervention targeted at caregiving dyads; (iv) published in English with an available full text. Correspondingly, articles that did not meet the outlined inclusion criteria were excluded. To ensure comprehensive coverage of the literature, reviews were included for citation tracking to identify further relevant studies. Figure 1 depicts a PRISMA flowchart displaying the decision process for study inclusion.

### 2.3. Data charting, summarizing, and reporting of results

The research team developed a standardized charting form using Microsoft Excel for data charting, informed by JBIs guidance (51). A quality appraisal of the included studies was not conducted, as this is not necessitated for scoping reviews (51). The results were synthesized in three steps:

1. Identifying psychosocial interventions: A detailed description of the included psychosocial interventions was extracted, including authors, publication year, the country in which the study was conducted, stated aim, study design, intervention characteristics, caregiving setting, nature of the caregiving relationship (formal/informal) and implementation/delivery method from each study. All authors reviewed the charting sheet, and VH and WQK piloted the data charting using 20% of the identified studies to ensure consistency in data extraction. After that, VH charted the remaining 80% of the data independently, verified by WQK upon completion.

2. Identifying intervention components: VH deductively coded the extracted data by mapping the identified intervention components onto the three core elements. WQK verified this process. No percent agreement or kappa coefficient was calculated in this process, rather, all intervention components were rigorously assessed using the operationalized enrichment core elements and the standardized charting form. Any disagreements were discussed until consensus was achieved.

3. Identifying enrichment categories: The identified intervention components were grouped within each core element to identify categories of intervention components that may contribute to enrichment in dementia caregiving relationships. These categories were discussed among all authors.

The narrative below provides an overview of the main intervention types and the component categories within each operationalized enrichment core element (summarized in Table 4).

## 3. Results

The database search resulted in 1,199 publications included for title and abstract screening, with an additional 65 articles from citation tracking of screened literature reviews. Following the full-text screening of 122 articles, 34 articles were included. The screening process is summarized in Figure 1.

### 3.1. Study characteristics

Twelve countries were represented in the studies conducted, of which the majority were in Europe (*n* = 19, including Belgium, Germany, Netherlands, Norway, Portugal, Sweden, and the United Kingdom). Other studies were conducted in Australia (*n* = 4), Canada (*n* = 3), Hong Kong (*n* = 1), and the US (*n* = 7). Two articles included multi-national studies (56, 57). Three research approaches were employed, including 12 quantitative, 11 qualitative, and 11 multi- or mixed-methods. Fifteen studies described interventions for community-dwelling caregiving dyads, while seventeen described interventions conducted in institutional settings. The final two studies included both settings. The sample size in each study ranged from three (one PLWD and two caregivers) to 1,515 participants (550 PLWD and 965 caregivers). The nature of the caregiving relationships was not necessarily dependent on the caregiving setting. Of the interventions conducted in institutional settings, two focused exclusively on the dyadic relationship of residents with dementia and their visiting family members (58, 59), while six articles reported on psychosocial interventions, including both nursing staff and family caregivers in social interactions with PLWD (60–65). All studies conducted in a community-based setting were focused solely on informal dyadic relationships with family members being the primary caregivers (*n* = 17; i.e., no studies reported on non-relatives). Table 3 summarizes the studies’ characteristics and the psychosocial interventions delivered.

**TABLE 3:** Study characteristics.

References, country	Methodology and study design	Participants *IC: Informal caregiver *FC: formal caregiver	Setting	Caregiving relationship	Intervention
Beentjes et al. (69), Netherlands	Quantitative, pilot RCT	PLWD: *n* = 59 IC: *n* = 59	Community-based	Informal	**Self-management resource:** Mobile application-based selection tool to support self-management and social participation
Bemelmans et al. (60), Netherlands	Multi-method, feasibility study	PLWD: *n* = 23 IC: *n* = 8 FC: *n* = 8	LTC setting	Formal *and* informal	**Social pet robot:** PARO, a baby-harp seal robot used for (i) therapeutic purposes for PLWD; (ii) facilitating daily care activities; (iii) supporting social visits
Bird (89), Sweden	Qualitative, case study	PLWD: *n* = 3 FC: *n* unclear	LTC setting	Formal	**Supervision and support project:** Organizational intervention involving (i) fortnightly staff support meetings with room to vent; (ii) more ownership for nurses in development and implementation of care plans for small groups of residents with dementia
Camic et al. (79), UK	Mixed-methods, pre/post explorative study	PLWD: *n* = 12 IC: *n* = 12	Community-based	Informal	**Art intervention:** Dyadic intervention involving art viewing, discussion and art-making for PLWD and their carers
Charlesworth et al. (71), UK	Quantitative, RCT	PLWD: *n* = 289 IC: *n* = 289	Community-based	Informal	**(1) Peer support** Career supporter Program (CSP); with volunteer carer supporters paired with a family carer **2) Reminiscence therapy** for both dyad members in groups: (Remembering Yesterday Caring Today; RYCT), including listening and communication skills for the carer
Chaudhury et al. (88), Canada	Multi-method, pre/post-test ethnographic study	PLWD: *n* = 10 FC: *n* = 17	LTC setting	Formal	**Environmental renovation:** Renewal and/or renovations of room and furniture to create a more responsive dining room (and home-like) environment for residents with dementia
Clair and Ebberts (58), USA	Quantitative, quasi-experimental pre/post-test	PLWD: *n* = 15 IC: *n* = 15	LTC setting	Informal	**Music intervention:** Music therapy program including singing, dancing, and rhythm playing. All participants were engaged in 10 min of each of (a) initial conversation; (b) singing; (c) ballroom folk or chair dancing; (d) rhythm participation using drums; (e) follow-up conversations
Crispi and Heitner (59), USA	Qualitative, single group post-test study	PLWD: *n* unclear IC: *n* = 29	LTC setting	Informal	**Reminiscence therapy:** A recreational program consisting of activity kits designed to benefit both the resident with dementia and the family carer during family visits. Kits were designed to stimulate memory and communicative ability, improve mood and facilitate family visits
Damianakis et al. (72), Canada	Qualitative and pilot study	PLWD: *n* = 12 IC: *n* = 27	Community-based *and* in LTC facilities	Informal	**Reminiscence therapy:** Multimedia biographies (MBs) to facilitate social interaction. MBs designed in collaboration with the PLWD, including videos, photos and stories. PLWD and family carers viewing the MBs together
Fritsch et al. (73), USA	Quantitative and observational study using an experimental design	PLWD: *n* unclear FC: *n* = 192	LTC setting	Formal	**Story telling groups:** a group storytelling program to encourage creative expression in PLWD and their carers. TimeSlips (TS) storytelling with a facilitator making stories based on residents’ pictures and their reflections around them, periodically reading it back to the participants as it progressed, to maintain the group’s focus and enthusiasm. Stories were often included in a facility’s newsletter or collated into books for families
Froggatt et al. (63), UK	Multi-method and cluster-RCT	PLWD: *n* = 32 IC: *n* = 20 FC: *n* = 97	LTC setting	Formal *and* informal	**Palliative care:** Namaste Care - creating a calm environment, a loving touch approach. Namaste Care was designed to ameliorate challenging symptoms to improve the quality of life and care at the end of life using “loving touch”, with touch thought to evoke an emotional response that leads to physical engagement
Goodall et al. (57), Norway, Belgium, Portugal, and Romania	Qualitative and explorative study	PLWD: *n* unclear FC: *n* = 8	LTC setting and one hospital	Formal	**Reminiscence therapy:** Technology-supported reminiscence therapy, including multiple stimuli in a dedicated room to facilitate dyadic interaction. SENSE-GARDEN, a room with technological solutions to deliver individualized interventions to PLWD, including family photographs, project films and images, surround sound music, factory stimuli, and exergame activities w/stationary bike accompanied by a film of a familiar place
Götell et al. (78), Sweden	Qualitative and ethnographic study	PLWD: *n* = 48 FC: *n* = 35	Geriatric Clinic, Special Care Unit	Formal	**Music intervention:** Music events including singing, active participation and movements. Music events incorporated biweekly with personnel singing and playing the guitar while supporting PLWD to move to the music and adapting music instruments for PLWD
Hagens et al. (61), Canada	Qualitative and Study design unclear	PLWD: *n* = 5 IC: *n* unclear FC: *n* unclear	LTC setting	Formal *and* Informal	**Reminiscence therapy:** Multi-sensory props to stimulate memories in poetry-making sessions, which thereafter were combined with photographs and objects meaningful to the PLWD to comprise Remembering Boxes used to facilitate communication
Hamel et al. (62), USA	Mixed-methods, convergent parallel design	PLWD: *n* = 18 IC: *n* = 8 FC: *n* = 6	LTC setting	Formal *and* informal	**Reminiscence therapy:** Memory matters (MM) Mobile tablet application containing text, audio clips and visual cues to facilitate reminiscence and cognitive stimulation
Jeon et al. (84), Australia	Qualitative and Case study	PLWD: *n* = 1 IC: *n* = 2 FC: *n* unknown	Community-based	Formal *and* informal	**Reablement Program:** Person-centered interdisciplinary program, I-HARP, including psychoeducation for carers and direct interventions by healthcare professionals designed to improve functional capacities in PLWD
Kihlgren et al. (80), Sweden	Qualitative and Study design unclear	PLWD: *n* = 5 FC: *n* unclear	LTC setting	Formal	**Carer training/education program:** “Integrity Promotion” to promote integrity among care recipients through improved interactions with dementia during morning care, through trust, autonomy, initiative, industry, identity, intimacy, generativity, and integrity
Kuot et al. (77), Australia	Qualitative and Pilot study	PLWD: *n* = 10 FC: *n* = 15	LTC setting	Formal	**Music intervention:** Personalized music listening intervention. Families of PLWD assisted in providing the lists of songs their relatives used to like, play or listen to before the onset of dementia. Staff facilitated each resident with dementia to listen to individualized digital music playlists daily
Lasrado et al. (56), UK and Sweden	Multi-method and prospective non-randomized feasibility study	PLWD: *n* = 43 IC: *n* = 43	Community-based	Informal	**Self-management resource:** Mobile application tool, DemPower, to support self-management in everyday life and enhance couple relationships through themes and activities
Leroi et al. (66), UK	Multi-method and RCT	PLWD: *n* = 76 IC: *n* = 76	Community-based	Informal	**Cognitive stimulation therapy (CST):** Manual-based individualized CST, CST adapted for Parkinson’s disease, sessions including discussion topics, word association games and creative tasks to facilitate positive discussion, enjoyable activities, affection and supportive feedback
McCallion et al. (64), USA	Quantitative and RCT	PLWD: *n* = 66 IC: *n* = 66	LTC setting	Formal *and* Informal	**Carer training/education program:** Family Visit Education Program (FVEP) including group sessions and individual family conferences, FVEP, aimed at improving the quality of interaction between family members and nursing home residents with moderate and severe dementia. FVEP designed to address (i) verbal communication, (ii) non-verbal communication, (iii) effective structuring of family visits
Nordheim et al. (81), Germany	Quantitative and RCT	PLWD: *n* = 108 IC: *n* = 108	Community-based	Informal	**Dyadic educational program:** Couples-based intervention, DYADEM, focusing on the dyadic relationship as the main resource by providing sessions containing information about dementia, couple communication, coping- and problem-solving strategies, network and activity analysis, counseling for living space adaptions and relaxation techniques
Orgeta et al. (67), UK	Mixed-methods and RCT with an embedded QUAL explorative study	PLWD: *n* = 356 (*n* = 22 in QUAL) IC: *n* = 356 (*n* = 22 in Qual)	Community-based	Informal	**Cognitive stimulation therapy (CST):** Individualized CST, iCST, consisting of themed activity sessions for caregiving dyads, including being creative, number games and art discussions
Palo-Bengtsson et al. (75), Sweden	Qualitative and phenomenological	PLWD: *n* = 6 FC: *n* unclear	LTC setting	Formal	**Music intervention:** Social dancing incorporated as a natural part of the carers’ regular duties and patients’ daily routine. Dances facilitated by musicians, ensuring residents had their favorite tunes sung and encouraging requests for their favorite music
Rahja et al. (85), Australia	Qualitative, descriptive study	PLWD: *n* = 5 IC: 10	Community-based	Informal	**Reablement program:** Dyadic intervention, COPE, to support physical and cognitive QoL of PLWD, and wellbeing of their carer. COPE included psychoeducation for carers to develop caregiving strategies around stress management, communication and involvement of PLWD in meaningful activities
Resnick et al. (82), USA	Quantitative and RCT	PLWD: *n* = 550 FC: *n* = 965	LTC setting	Formal	**Carer training/education program:** “Function Focused Care” utilizing person-centered care approaches to engage PLWD in physical activity to optimize care interactions and avoid custodial care
Ritchie et al. (70), UK	Qualitative and explorative with realistic evaluation design	PLWD: *n* = 4 IC: *n* = 4	Community-based	Informal	**Animal-assisted intervention:** Employment of dogs trained to live at home with a dementia caregiving dyad to provide personalized support to them, supported by training sessions with the couple and the dog
Spector et al. (86), UK	Quantitative and RCT	PLWD: *n* = 50 IC: *n* = 50	Community-based	Informal	**Cognitive behavioral therapy (CBT**): Manual-based dyadic interventions focused on building collaborative relationships, psychoeducation and implementation strategies, addressing the interaction between people’s thoughts, feelings and behavior
Tamplin et al. (76), Australia	Multi-method and pre/post-test feasibility study	PLWD: *n* = 12 IC: *n* = 12	Community-based	Informal	**Music intervention:** Singing therapy, Remini-Sing, for PLWD and their family carers, including social interaction and peer support, to improve or maintain personal relationships, social engagement and emotional wellbeing. Remini-Sing follows a person-centered, strength-based and process-oriented approach
van Weert et al. (68), Netherlands	Quantitative and quasi-experimental pre/post-test	PLWD: *n* = 121 FC: *n* = 121	LTC setting	Formal	**Multi-sensory stimulation (MSS):** communication tool through positive affective communication. MSS provides pleasurable sensory stimulation with light, sound, feeling and taste tailored to the needs of the PLWD
Williams et al. (83), USA	Multi-method, single-group pre/post-test feasibility study	PLWD: *n* = 15 IC: *n* = 15	Community-based	Informal	**Carer training/education program:** Manual-based communication intervention, CARE, to facilitate relationship-focused communication in caregiving dyads through information and communication strategies tailored to each of the dyads’ needs
Windle et al. (65), UK	Mixed-methods and pre-post longitudinal mixed-methods	PLWD: *n* unclear IC: *n* = 88 FC: *n* = 58	LTC setting *and* Community-based	Formal *and* informal	**Art intervention:** Dyadic intervention involving art viewing, discussion and art-making, adjusted to the degree of cognitive impairment in the group
Woods et al. (74), UK	Quantitative and RCT	PLWD: *n* = 487 IC: *n* = 486	Community-based	Informal	**Reminiscence:** Joint reminiscence groups, “Remembering Yesterday, Caring Today” (RYCT), with sessions including art, cooking, physical re-enactment of memories, singing and oral reminiscence. Developed for PLWD and their family carers, including guidance for carers to allow PLWD to respond and value their contribution
Yu et al. (87), Hong Kong	Quantitative and RCT	PLWD: *n* = 103 IC: *n* = 103	Community-based	Informal	**Empowerment program:** strength-based and empowerment approach, D-StEP-MCI to address complex dyadic needs. Focused at (i) motivating active participation of the dyads; (ii) identifying collective coping strengths; (iii) building upon effective self-care; (iv) facilitate dyadic skill acquisition in adapting to functional loss

### 3.2. Types of psychosocial interventions

A wide array of interventions containing elements contributing to the operationalized definition of enrichment was identified. The interventions were categorized into three groups: (1) engagement in dyadic activities; (2) carer education or training; and (3) restructuring the caregiving framework around PLWD.

#### 3.2.1. Supporting dyads to engage in shared activities

Most studies reported interventions (*n* = 22) which involved supporting caregiving dyads to participate in social activities (either supported by objects for shared attention or structured social sessions), cognitive and multisensory stimulation and self-management. Carer-provided stimuli for PLWD was a central means to support social participation, either through cognitive stimulation therapy (CST) (66, 67) or in dedicated rooms, the latter including multisensory stimulation to improve caregiving in institutional settings through meaningful social interactions in the caregiving dyads (57, 68). Bemelmans et al. (60) reported on an intervention involving the social pet robot, PARO, to elicit social engagement and facilitate interactions between dyads. Two interventions employed mobile applications to promote self-management and social participation in community-dwelling caregiving dyads (56, 69), while another intervention introduced a dementia assistance dog to support dyads at home (70).

Seven interventions included reminiscence therapy or reminiscence-based activities encompassed in a multi-component intervention (59, 61, 62, 71–74), either one-to-one (59, 61, 62, 72) or in groups (71, 73, 74). Two interventions utilized digital devices to promote reminiscence (62, 72), while four facilitated reminiscing using a collection of objects meaningful to the PLWD (59, 61) or through group conversations (71, 73, 74). Five of the remaining interventions implemented music, focusing mainly on social dancing (75), singing therapy (76), personalized music lists (77), or active music sessions combining singing, movement and playing instruments (58, 78). Two interventions involved engaging a professional artist to facilitate structured group sessions for community-dwelling dyads, involving group-based art-viewing, discussion and art-making (65, 79).

#### 3.2.2. Interventions including carer education or training

The second category encompassed ten interventions covering carer training either as a single-component intervention (64, 80–83) or using educational modules as one of the multicomponent interventions (63, 84–87). Interventions focusing solely on carer education and training included modules to promote integrity for PLWD (80), enhancing family visits to nursing homes (64), dyadic communication training (81, 83) and training to support PLWD’s functional abilities (82). Where education and training were part of multicomponent interventions, they focused on re-ablement (84, 85), empowerment (87), cognitive behavioral therapy (CBT) (86), and palliative care (63). The emphasis of these interventions was on promoting emotional health, physical strengths or cognitive capabilities in PLWD.

#### 3.2.3. Restructuring the caregiving framework around PLWD

The final category contains only two interventions focused on the caregiving environment. In one study, the physical environment of the dining room in a nursing home was renovated to create a sense of homeliness for PLWD (88). In another study, the social aspect of care provision was changed to support care staff to take more ownership in developing and implementing care plans for their residents (89).

### 3.3. Intervention components falling under the concept of enrichment

**FIGURE 2:**
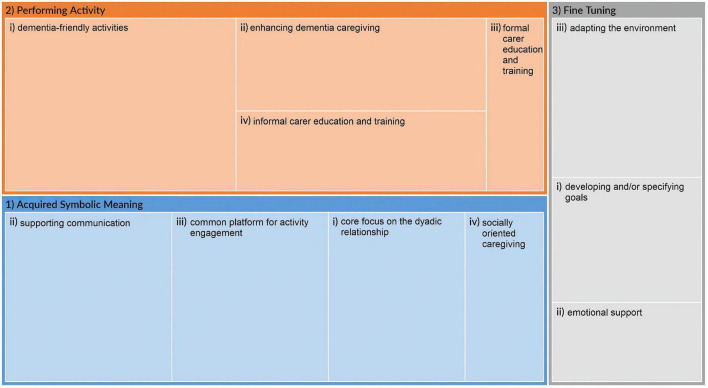
Treemap of identified categories within each enrichment core element.

Many interventions contained components contributing to several core elements of enrichment in multiple ways, resulting in some overlap of subcategories. Table 4 summarizes the articles’ identified intervention components contributing to enrichment. The synthesized results are organized and presented categorically according to each core element of enrichment. Within each core element, categories of intervention components were identified; The treemap below displayed these categories and their relative sizes within each core element (Figure 2).

**TABLE 4:** Identified intervention elements contributing to enrichment in dyadic relationships.

References	Acquired symbolic meaning	Performing activity	Fine tuning
Beentjes et al. (69)	1d:**^1^** Focuses on providing meaningful activities for both dyad members (iii)**^2^**	2a: Focuses on facilitating social participation for both dyad members (i)	3b: Training provided to carer to take on a supportive role (iii) 3c: Focuses on supporting independent living (iii) 3c: Focuses on the strengths of PLWD by providing a failure-free environment (iii)
Bemelmans et al. (60)	1a: Provides a point of joint attention for social interaction between the dyad members (iii) 1c: Supports positive experiences in caregiving by stimulating more attractive visits (i, iii)	2a: Focuses on supporting daily care activities for formal carers (ii) 2a: Focuses on facilitating social participation for PLWD (i)	3a: Specifies individualized care/therapeutic goals for the individual with dementia (i)
Bird (89)			3b: Training for staff to adapt care to symptoms of dementia in the individual 3b: Provides emotional support to staff to cope with symptoms of dementia (ii)
Camic et al. (79)	1a: Provides a point of joint attention for social interaction between the dyad members (iii) 1b: Supports topics introduction in the caregiving dyad through structured art-sessions (ii)	2a: Facilitates social participation through activities specifically adapted to dementia caregiving dyads (i)	
Charlesworth et al. (71)		2a: Facilitates social participation through activities specifically adapted to dementia caregiving dyads (i) 2b: Facilitates reminiscence on shared memories in the dyads (i) 2c: Training of carer in listening and communication skills with PLWD (iv) 2d: Care supporters with specific skills in supporting family carers of PLWD to cope involved (ii)	
Chaudhury et al. (88)			3a: Creates a more responsive dining environment for PLWD to provide a more home-like feeling as a base for dyadic interaction (iii)
Clair and Ebberts (58)	1b: Supports topics introduction through structured music sessions (ii) 1c: Focuses on facilitating positive experiences in the caregiving dyads through conversations and shared social activities using music (i, iii)	2a: Focuses on facilitating social participation through activities specifically adapted to dementia caregiving dyads (i)	
Crispi and Heitner (59)	1a: Provides a point of joint attention for social interaction between the dyad members (iii) 1c: Supports positive social interactions through activity kits designed to be enjoyable for both dyad members (i, iii)	2a: Facilitates social participation through activities specifically adapted to dementia caregiving dyads (i)	3a: Adapts the activities to the cognitive and communicative capabilities of PLWD (iii)
Damianakis et al. (72)	1a: Provides a point of joint attention for social interaction between the dyad members (iii) 1b: Supports topics introduction using personalized multimedia biographies (ii)	2a: Facilitates social participation through activities specifically adapted to dementia caregiving dyads (i)	
Fritsch et al. (73)		2a: Provides activities specifically adapted for carers to engage PLWD (i, ii)	3a: Focuses on the strengths of PLWD by providing a failure-free environment (iii)
Froggatt et al. (63)	1a: Focuses on touch triggering engagement and connection between residents and carers (iv) 1c: Focuses on caregiving beyond task-oriented care building on familiarity, reassurance, trust and mood (iv) 1d: Focuses on enabling meaningful relationships to form between providers and residents (i)	2a: Focuses on having the same person provide care each session by incorporating one-to-one interaction into an activity (ii) 2a: Provides regular and structured access to social and physical stimulation for residents with advanced dementia (i, ii) 2c: Equips carers with skills to work with people with advanced dementia (iii)	
Goodall et al. (57)	1a: Incorporates a dedicated room designed to facilitate social interaction between dyad members (iii, iv)	2a: Facilitates social participation through individualized stimuli such as family photographs, music, scents, exergaming with film of familiar places (i, ii)	
Götell et al. (78)		2a: Facilitates social participation through structured music sessions including instruments designed to be played by PLWD and encouragement and playing familiar songs (i, ii)	
Hagens et al. (61)	1a: Focuses on facilitating social interaction between the dyad members through structured reminiscence sessions to create personal poetry together (iii) 1b: Focuses on supporting dyadic communication by creating remembering boxes which potentially can enhance communication (ii)	2a: Focuses on engaging PLWD in social activities through personalized reminiscence props (i) 2c: Trains carer in appropriate situations to use the remembering boxes to facilitate social interactions (ii–iv)	
Hamel et al. (62)		2a: Engages carers in structured social sessions together with PLWD using reminiscence tools (i)	3a: Facilitates cognitive stimulation through reminiscence activities using supportive tools specifically designed for PLWD (iii)
Jeon et al. (84)	1b: Encourages carers to communicate with PLWD and to focus on openness in the dyadic communication (i, ii)	2c: Family psychoeducation for carers including information on dementia, coping strategies, reablement and person-centered care principles, focus on own needs (iv)	3a: Comprehensive multidisciplinary assessment of functional abilities to identify goals and strategies in the caregiving dyad (i) 3a: Intervention elements including cognitive rehabilitation, energy conservation and task simplification strategies, anxiety and pain relief management, problem solving, modification, meetings and follow-up (iii) 3b: Encourages carers to facilitate opportunities for PLWD to follow interests through family activities and use of home care support (ii) 3c: Interdisciplinary plan tailored to meet PLWD’s needs to enhance self-care ability (i)
Kihlgren et al. (80)		2c: Provides staff training focusing on how to promote trust, autonomy, initiative, industry, identity, intimacy, generativity, and integrity in different care activities (ii, iii)	
Kuot et al. (77)		2a: Carers engaged in creating individualized musical playlists for their residents with dementia (i)	
Lasrado et al. (56)	1a/1b: Focuses on facilitating dyadic social interactions through topics introduction (ii) 1b/1c/1d: Activities focusing on meaningful activities and relationships, communication and emotions (i–iii) 1d: Focuses on enhancing couples’ relationships in dementia (i)	2a: Focuses on providing shared activities for dementia caregiving dyads with a self-management resource specifically designed for them (i)	3b: Supports the dyad members to adapt and cope with changing abilities and limitations through opportunities for reflection and active participation encompassed in activities (ii)
Leroi et al. (66)	1a: Focuses on facilitating positive discussion, enjoyable activities, affection, supportive feedback and a focus on opinions rather than facts between dyad members (i–iii)	2a: Provides activities specifically designed for dementia caregiving dyads (i) 2c: Provides training for carers in how to deliver individualized CST (iv)	3a: CST activities varying in complexity, matched and adapted to suit the needs of the recipient (iii)
McCallion et al. (64)	1a: Family visit education program focusing on (non)verbal communication and how to structure social interactions during visits (ii) 1a: Focuses on facilitating dyadic communication during visits (ii)	2c: Carer education program including information on strain, strategies and dementia’s impact on social interaction (iv) 2c: Includes therapeutic observation of social interaction with interactive feedback sessions with family members (iv)	3b: Focus on helping participants to become more familiar with the cognitive, affective, and behavioral responses of PLWD (ii)
Nordheim et al. (81)	1a: Facilitates dyadic interaction by focusing on both dyad members contributing equally with their experiences, needs and wishes of during the sessions (i) 1b: Therapy focusing on couple communication (ii) 1d: Places focus on at the dyadic relationship as a resource (i)	2c: Carer training by providing information about dementia to carers (iv) 2d: Facilitates shared social sessions by involving both dyad members as far as possible (i)	3a: Support program focusing on personal, dyadic and environmental resources (ii) 3b: Focuses on strengthening the dyadic relationship as main resource in coping and living with dementia including activity analysis, coping and problem-solving strategies, adapting living space and relaxation techniques (i, iii)
Orgeta et al. (67)	1b: Focuses on supporting dyadic communication through structured discussions with PLWD prompted by family carers (ii)	2a: Focuses on promoting social participation by offering activities specifically designed for dementia caregiving dyads (i)	
Palo-Bengtsson et al. (75)	1a: Facilitates social interaction between dyad members by encouraging participation in PLWD by requesting their favorite music (iii, iv)	2a: Promotes shared activities for the caregiving dyads through incorporating dancing as a natural part of the carer’s duties and the patients’ daily routine (i, ii)	
Rahja et al. (85)	1b: Focuses on support dyadic communication by equipping carers in communication techniques (ii)	2c: Focuses on educating and advising carers with strategies including how to encourage PLWD to participate in valued activities (i, iv)	3b: Provides carers with strategies around stress management, identifying areas of concern and problem-solving different approaches around modifying (carer) communication and the home environment (i, iii)
Resnick et al. (82)	1a: Focuses on facilitating social interaction between dyad members by optimizing care interactions (iv)	2c: Carer education program to avoid custodial care and engage residents with dementia in physical activity (ii, iii)	3c: Focuses on building PLWD strengths and capacities by facilitating carers to enable residents with assistive devices facilitating independence or approaches to maintain or optimize functional independence (i, iii)
Ritchie et al. (70)		2a: A dementia assistance dog specifically trained to live at home with a dementia caregiving dyad (i, ii)	
Spector et al. (86)		2b: Focuses on activity-based therapies involving both dyad members in cognitive-behavior therapy (iv) 2c: Provides carer psychoeducation about CBT, information about dementia, self-monitoring, developing individualized formulation and identifying goals (iv)	3a: Focuses on building assets by helping carers support PLWD in implementing strategies (i) 3b: Focuses on supporting dyad members adapting and coping according to their needs and strengths and addressing interpersonal difficulties between the carer and PLWD (i, ii)
Tamplin et al. (76)	1a/1b: Focuses on facilitating social interaction between dyad members through group singing, communication support, and encouraging social engagement (ii, iii) 1d: Focuses on maintenance of positive and meaningful social relationships with singing as strategy to improve/maintain the personal relationships (i)	2a: Provides a shared activity appropriate to dementia caregiving dyads through therapeutic group singing (i) 2b: Provides activity-based therapy in the form of music therapy for both dyad members in groups (ii)	3c: Focuses on strengths and capabilities of PLWD by being person-centered, strengths-based and process-oriented, rather than product or performance oriented (iii)
van Weert et al. (68)	1a: Focuses on facilitating social interaction between the dyad members by being a means of making contact and aims for pleasurable sensory experiences, tailored to the needs PLWD (iii, iv) 1b: Focuses on supporting dyadic interaction by serving as a tool to communicate with people with severe dementia (ii, iv) 1d: Focuses on maintenance of positive and meaningful social relationships through social conversation not related to nursing activities, showing agreement and understanding (i, iv)	2c: Provides carer education including attitudes toward (non)verbal communication and the need for (non)verbal attentiveness (iii) 2c: Focuses on developing awareness of PLWDs’ physical, social and emotional needs (ii, iii)	3a: Focuses on PLWDs’ potential by avoiding giving useless cognitive information, avoiding testing remaining cognitive knowledge and avoiding to correct residents’ subjective reality (i, iii)
Williams et al. (83)	1b: Focuses on increasing facilitative communication in the carer and sociable communication in PLWD (ii) 1d: Focuses on using communication to decrease isolation and to strengthen/maintaining the caring relationship (i, ii)	2c: Provides carer education on communication issues including overview of communication strategies, responding to conflict, and understanding non-verbal communication (iv)	3b: Equips carer with coping and adapting strategies, supportive follow-up meetings, and emotional support by providing room to express thoughts, feelings and preferences (ii)
Windle et al. (65)		2a: Focuses on providing shared activities appropriate for dementia caregiving dyads through art-viewing activities and art-making adjusted according to the varying degrees of cognitive impairment (i)	
Woods et al. (74)		2a: Provides shared activities for the caregiving dyad to engage in together through activities (i) 2c: Provides carer education for reminiscing with PWLD with carers guided to allow PLWD to respond and to value their contribution (iv)	
Yu et al. (87)	1a: Focuses on facilitating social interaction between dyad members through motivating active participation of the care dyads (i, iii)		3a: Supports identification of dyad members’ individual and collective strengths in coping with cognitive decline, building on effective self-care or caregiving situations, or facilitating acquiring new skills and adapt to functional loss (ii) 3b: Facilitates identification of challenges for maintaining independence, engagement and stress adaptation (i) 3c: capitalize identified abilities of PLWD and monitor level of goal attainment (i)

#### 3.3.1. Acquired symbolic meaning

When analyzing the interventions included in this review, elements contributing to this enrichment core element were categorized into four categories: (i) core focus on the dyadic relationship; (ii) supporting communication; (iii) common platform for activity engagement; and (iv) socially oriented caregiving. *Core focus on the dyadic relationship:* Nine studies described interventions with a core focus on supporting dyadic relationships. These interventions were targeted at improving or maintaining the dyadic relationship (76, 81, 83), facilitating positive experiences (58, 60, 68), promoting connectedness in the dyad (56, 63) or enhancing the quality of social interactions (64). *Supporting communication:* The largest category (as depicted in Figure 2) of interventions’ contribution to “acquired symbolic meaning” was communication support, either using tools to help caregivers identify topics of conversation (56, 61, 68, 72), facilitating structured discussions (58, 66, 67, 79, 81) or providing training in verbal and/or non-verbal communication techniques (66, 81, 83, 85). *Common platform for activity engagement:* Several interventions provided a common platform for social interaction between dyad members. By providing a point of joint attention between the dyad members (59, 60, 72, 79), the interventions created a framework within which they could engage in meaningful activities together (56, 57, 69, 76). Regardless of whether the interventions aimed to stimulate positive social interactions (59–61, 66) or empowerment (56, 87), the fact that the focus was on the social aspects themselves and not on regular caregiving duties (63, 68, 75) provided structure to the social interactions. *Socially oriented caregiving:* Some interventions (*n* = 6) described a shift from “passive” caregiving responsibilities to a more socially oriented approach (82) of enabling meaningful relationships to form between care staff and PLWD (63, 68), encouraging openness in the dyadic relationship (84) and engaging PLWD in social interactions in the caregiving routine (75, 82).

#### 3.3.2. Performing activity

Thirty-one interventions contained elements that contributed to enrichment within the core element of “performing activity”, and could be divided into four categories: (i) dementia-friendly activities; (ii) enhancing dementia caregiving; (iii) formal carer education and training; and (iv) informal carer education and training. *Dementia-friendly activities:* Most interventions engaged both dyad members in dementia-friendly activities, and as Figure 2 shows, this category was the most-represented category of all intervention components across all three core elements. These intervention components involved individualized social and physical stimulation for PLWD (57, 59, 61, 62, 66, 67, 72, 77) to engage both dyad members in social interactions. *Enhancing dementia caregiving:* Interventions which, in one way or another, could facilitate caregiving activities (60, 63, 70, 76, 86). This included emphasis on having the same person providing one-to-one care for PLWD (63), supporting daily (care) activities using a live or robotic pet (60, 70) and activity-based therapies involving both dyad members (76, 86). *Formal carer education and training:* Despite being the smallest category according to the treemap (Figure 2), several approaches were used to educate and train staff in institutional settings. These included education on providing optimized care through improved interactions (63, 80, 82), awareness of the physical, emotional, and social needs of PLWD (68), engaging PLWD in social activities using supportive tools (64, 68) or person-centered care approaches (82). *Informal carer education and training:* In contrast to approaches for staff, the education for informal caregivers was focused on supporting them to adapt and adjust to the dementia diagnosis. These included education on coping strategies (81, 84), communication and listening (64, 66, 71, 81, 83), conflict solution (83), and socially engaging their loved ones with dementia through supportive tools (61) or skill acquisition (64, 66, 74, 85, 86).

#### 3.3.3. Fine tuning

Although the intervention components were not as strongly represented within “fine tuning” as the two other core elements, three categories were identified: (i) developing and/or specifying goals; (ii) emotional support; and (iii) adapting the environment. *Developing and/or specifying goals:* Several intervention components included supporting one or both dyad members to formulate individualized goals and strategies in formal and informal caregiving relationships, to support their adaption and coping with symptoms of dementia and changing needs and capabilities (60, 81, 84–87). These included helping dyad members identify their individual and collective strengths (81, 87), areas of concern or difficulties (85, 86) and monitoring goal attainment (84, 87). Some involved specialist assessments of functional abilities and activity analysis (81, 84) or programs developed by specialists such as clinical psychologists (86) or occupational therapists (85). For community-dwelling dyads, intervention components also involved equipping them with skills to manage stress, anxiety and pain (84, 85), problem-solving and task simplification (85, 86). Maintaining or optimizing independence was an underlying theme in many of the goals specified through self-care (84), problem-solving strategies (81, 84, 85) and acquiring new skills in adapting to functional loss (87). Independence for PLWD or community-dwelling dyads was also sought through digital self-management tools (56, 69) or care approaches (82, 87). *Emotional support:* Several interventions emphasized providing emotional support to help carers become more familiar with common symptoms and behavior in dementia (64, 86), providing opportunities for reflection and active participation (56, 83, 84, 89), using the relationship as a resource for coping (81, 86, 87), and to address challenges in the dyadic relationship (86, 89). This support was mainly directed at informal caregivers (56, 64, 81, 83, 84, 86, 87), but one intervention included emotional support for staff in a nursing home (89). *Adapting the environment:* Some interventions described environment adaptation to support the relationships between PLWD and their caregivers, such as the physical revamping of the dining space in an LTC setting to create a more responsive environment (88), or adapting the homes of community-dwelling caregiving dyads (81, 84, 85), Creating “failure-free” social environments to support engagement in group activities (73) or using virtual tools supporting social interaction were important strategies to accommodate PLWD (62, 69). Finally, activity grading and task simplification were also present when adapting the environment to tailor to the cognitive, communicative and physical abilities of PLWD (59, 66), focusing on potential and capabilities rather than performance (68, 76), as well as energy conservation (84).

## 4. Discussion

The objective of this review was to scope the body of literature on what constitutes enrichment in dementia caregiving by mapping components of psychosocial interventions onto our operationalized core elements of enrichment. To the best of our knowledge, this scoping review is the first to systematically identify and synthesize existing evidence on psychosocial interventions that facilitate enrichment in dyadic caregiving relationships in dementia. Most included studies were community-based or conducted in LTC facilities and mainly provided shared activities for the dyads, carer education interventions and structural change to the environment around PLWD.

Overall, our findings indicate an important distinction between formal and informal caregiving relationships: In formal caregiving relationships (i.e., between PLWD and paid caregivers), interventions were mainly directed at changing the care provision, shifting focus from pure custodial care to caregiving with space for the social element in the dyadic interactions. Whether the intervention type involved carer education/training (80, 82), restructuring the physical or organizational environment (88, 89) or engaging dyad members in shared activities (57, 68, 73, 75, 77, 78), the interventions created dedicated space for building a relationship between caregiver and care recipient, where such space otherwise is limited. In contrast, interventions targeting informal caregiving relationships appeared to support relationship sustenance by mitigating some challenges that might follow a dementia diagnosis. These interventions contained components emotionally supporting the dyad members to adjust and cope with the diagnosis (64, 71, 81, 83, 86), facilitating positive social interactions (58, 59, 61, 66, 67, 72, 74, 76, 79) or empowering them through self-management (56, 69, 70, 85, 87). These findings highlight the importance of supporting formal caregivers to enrich caregiving relationships. As such, future interventions may consider dedicating resources, such as protected time (90, 91), to support positive social interactions. Similarly, enrichment in informal relationships, where informal caregivers constantly have to adapt to the changing relationship dynamics alongside the progression of dementia, may be generated through interventions that facilitate positive social interactions or support dyads to build on collective and individual strengths.

Within the first core element, “acquired symbolic meaning”, intervention components contributing to enrichment had one important thing in common, regardless of the type of relationship: They all, in some way or another, facilitated dyadic communication, either explicitly (i.e., the category *supporting communication*), or indirectly through communication-enhancing mechanisms (i.e., the category showing a *core focus on dyadic relationships; common platform for activity engagement;* or *socially oriented caregiving*). Additionally, as shown in Figure 2, communication support was the most-represented category within “acquired symbolic meaning”. Considering the communication challenges that follow a dementia diagnosis, communication support seems essential to enrichment in dementia caregiving dyads. Eggenberger and colleagues’ systematic review found that communication skills in dementia care improve the life and wellbeing of PLWD cared for both in the community and institutional settings (92). They also found that formal caregivers reported a greater feeling of control and joy from opportunities to learn more about the patients in their care (92). However, the practicalities of supporting PLWD through communication are rarely mentioned in research (93), and socially oriented care through such day-to-day communication should receive greater attention.

Intervention components falling under the core element of “performing activity” were mostly contained within dementia-friendly activities (such as individualized social and physical stimulation for PLWD) or carer training and education. While dementia-friendly activities are gaining momentum in dementia research on social health (30, 94–96), this review showed that relatively few studies explicitly focused on supporting caregiving dyads’ relationship. Considering the reciprocal influence dyad members have on each other (97, 98), interventions focusing on dyads seem more effective than interventions focusing separately on each individual (17, 98). As such, to generate enrichment in caregiving relationships, developers of psychosocial interventions may consider including both dyadic members on equal terms to enhance dyadic wellbeing (17). A common intervention in dementia care often includes caregiver training and education that is often focused on one dyadic member (e.g., on the caregiver). Such training and education programs may increase effectiveness by including an additional focus on the relational aspect of the caregiving dyad.

Intervention components contributing to the final core element of enrichment, “fine tuning”, primarily targeted informal caregiving relationships through interventions supporting community-dwelling caregiving dyads to adapt and cope with dementia through educational modules and emotional support. These categories of fine tuning deserve greater attention in dementia research since relationship quality is recognized as a significant factor influencing the health and wellbeing of both caregiver and care recipient, consequently impacting their ability to live together at home (76). Although entirely different conditions govern formal caregiving, one could argue that the importance of “fine tuning” is no lower once a person with dementia transfers to institutional care. However, few included studies reported on interventions delivered in institutional settings adapting an activity or environment to support PLWD’s participation in valued activities. Some examples were identified, such as creating a more responsive dining environment for dyadic interaction (88), story-telling groups (73), or educating staff to adapt and optimize interactions in caregiving (68, 82, 89). With research indicating that nursing home residents with dementia are involved in few social activities (99–102), there is a need for more interventions to support “fine tuning” in formal caregiving to facilitate shared social activities that might enrich the caregiving dyad. Moreover, as indicated by the third category of psychosocial interventions containing only two studies, more interventions are needed focusing on accommodating the physical environment to create room for enrichment.

Whether the interventions contained components that fell under one, two, or all three core enrichment elements, there was a vast array of different approaches that may enrich the dyadic relationship and thereby enhance the relationship quality through different mechanisms. Therefore, interventions aiming to enrich dyadic relationships are inherently related to relationship-centered care strategies, as such interventions may lead to relationship gains (29, 41). With research suggesting that enriching activities can provide a vehicle to maintain or strengthen dyadic relationships and enhance positive outcomes for both caregiver and care recipient (103), intervention components identified in this scoping review may further contribute to developing research-centered approaches. In dementia research, influential theories have traditionally leaned toward stress-coping models that focus on burden and strain (9, 104, 105). There is an ongoing shift toward a more positive dementia discourse, with increasing attention to the social health of PLWD and their caregivers (30, 31, 106, 107). Nevertheless, there is a need to conceptualize this focus (28, 108). Although not previously applied to dementia research, the model of Cartwright and colleagues offers a broad inclusion of different typologies of dyadic relationships while focusing on positive aspects of caregiving. The application of theoretical frameworks appropriate for dyadic processes has been emphasized as essential to expand our understanding of outcomes in dementia research (17), as they might lead to innovative approaches in working with caregiving dyads (108). As such, we believe that the model of Cartwright and colleagues may provide a useful framework when developing, implementing and evaluating psychosocial interventions taking on a relationship-centered approach.

### 4.1. Implications for future research

This study reviewed and clarified intervention components that may contribute to enrichment, which might provide a groundwork for future research aiming to promote positive caregiving experiences through enrichment by supporting relationship sustenance among dyads. Enrichment in interventions targeting formal caregiving relationships seems to require space dedicated to enabling social interactions beyond custodial care. Considerations such as protected time and explicit training on socially oriented care may be necessary (109–111) to allow positive relationships to emerge and grow. Interventions targeting informal dyads, on the other hand, may consider building on the dyads’ shared history and sustain or enhance their caregiving relationship by including components that may circumvent the challenges that follow when living with dementia, such as emotional support, coping and management strategy training or dementia-friendly shared activities.

Regardless of the relationship type, communication seems essential to contributing to enrichment in dyadic relationships interactions, calling for increased focus on communication skill acquisition and support in dementia research and practice. Naturally, the type of caregiving relationship constitutes a major contextual factor influencing the “process of endowing caregiving with meaning or pleasure” for both dyad members. Although the interventions shared many similarities in how their components contributed to enrichment, the nature of the caregiving relationship will unquestionably influence how the three core enrichment elements can be achieved. The same is true for the *outcomes* of enrichment in terms of impacting the dyad members and their relationships. Research suggests that the relational nature influences the relationship quality and the experiences of both dyad members living with dementia (15). Cartwright and colleagues included contextual factors and enrichment outcomes in their theoretical model (29). Future research into enrichment in dementia caregiving can therefore utilize this model and further extend it when focusing on outcomes of enrichment, as well as contextual influences.

### 4.2. Strengths and limitations

There are several strengths of this review. First, an established methodological framework was used to guide the conduct of the review. Next, there is no existing definition of enrichment in dyadic relationships; to minimize the potential subjectivities, this scoping review leveraged and built upon the model by Cartwright et al., using empirical research and the spearheaded work of the INTERDEM Social Health Taskforce (30–34). This enabled the concept of enrichment to be operationalized and systematically applied during the construction of the search strings, data screening, charting and synthesis. Next, the initial search strategy was refined and further developed in collaboration with an expert research librarian with extensive knowledge and experience in literature reviews. Finally, at least two independent reviewers were involved in the screening, charting, and data synthesis stages. A third reviewer was involved in resolving any disagreements.

There are also limitations to this review that must be acknowledged. Since enrichment has not been defined previously, there is a chance that relevant studies are not yielded from the current search strategy. Despite the operationalization of the concept of enrichment guiding the inclusion criteria, articles using different terms than those contained in our search strategy may not be identified. A systematic citation tracking of identified reviews was also conducted to minimize this risk. Nevertheless, other relevant studies might have been missed as only peer-reviewed publications in English were included. A final limitation that must be considered is that this review did not look into the outcomes of the psychosocial interventions. The realized individual or relational gains (or the lack thereof) following psychosocial interventions containing enriching components fall outside the scope of this review and are not synthesized. However, with no existing framework to follow in systematically promoting enrichment in the context of dementia, we consider this review as the first step in the development of an extensive groundwork focusing on the positive aspects of dementia caregiving.

## 5. Conclusion

This scoping review proposed taking on a relationship-centered approach using and extending a theoretical framework for enrichment to develop and promote psychosocial interventions supporting dyadic relationships in dementia caregiving. By charting the evidence of existing psychosocial interventions that may enrich relationships between caregiving dyads, we identified intervention components that may contribute to such enrichment. Interventions aiming to enhance formal caregiving relationships should focus on providing space for positive social interactions and room for relationships to build and grow. Interventions targeting informal caregiving dyads, on the other hand, need to consider the existing relationship when facilitating positive social interactions and provide support in coping and managing the changing relationship dynamics a dementia diagnosis might bring. Whether the caregiving relationship is formal or informal, dyadic communication support and skill acquisition seem vital in laying the groundwork for generating enrichment in the caregiving relationship. Findings from this review may inform the planning and development of enrichment interventions to improve or maintain dyadic relationships in dementia caregiving, which may ultimately lead to beneficial outcomes.

## Data availability statement

The original contributions presented in this study are included in the article/Supplementary material, further inquiries can be directed to the corresponding author.

## Author contributions

The review was conceived and planned by VH in conjunction with WQK and DS. An initial literature search strategy was developed and conducted by VH based on theoretical and empirical literature, which was thereafter revised and refined in collaboration with an expert research librarian. VH and WQK thereafter screened titles/abstracts and full-text articles for eligibility independently. Discrepancies between VH and WQK was discussed until consensus was achieved, and any discrepancies in the full-text screening phase was resolved by DS. VH and WQK independently piloted 20% of the data extraction before VH completed the remaining 80%, verified by WQK. The charting and analysis of extracted data was conducted by VH, again verified by WQK and discussed with DS. VH wrote the first draft of the manuscript, which was critically reviewed by WQK and DS, and the drafts were thereafter developed in an iterative process through joint discussions between all three authors. All authors agreed on the final manuscript submitted for publication.
