# Supplementary material for: Enrichment of dementia caregiving relationships through psychosocial interventions: A scoping review
Source: Front Med (Lausanne). 2023 Jan 5;9:1069846. doi: 10.3389/fmed.2022.1069846 (PMC9849912; doi:10.3389/fmed.2022.1069846)
Supplement: Supplementary file 1 [file Table_1.PDF]

Supplementary Material

1 Operationalization of enrichment in dementia and focus of psychosocial interventions to generate enrichment.

**Core elements: Original definition**	**Description and contextualized to dementia according to literature and empirical findings (Data source)**	**Operationalization**	**Focus of interventions to generate enrichment**
**Acquiring symbolic meaning:**<br><br>the significance, value or intent of an event or an object. The symbolic property reflects meaning that transcends the utility of the given object or event	The emotional aspect for a dyad member of being involved with an object or activity, with the symbolic meaning derived from the object/activity in question being in center of the dyadic relationship.<br><br>- the act of being occupied or involved in meaningful activities (1, 2)<br>- social interactions and having social ties and relationships, which are meaningful to the PWLD (1-3)<br><br>- positive experiences made together as a couple (1, 2)<br>- sustaining a sense of togetherness/connectedness (1, 2)<br>- enhanced communication (2, 3)<br>- learning more about the PLWD (capabilities, history, interests) (2)<br>- having a shared activity appropriate to dementia (1, 2)	Shared activities considered meaningful to the dyad members, that facilitate positive relationship gains (i.e., relationship quality, togetherness, closeness, connectedness), such as positive social interactions, improved communications, and shared activities appropriate to a dementia caregiving context	a. Facilitate social interaction between the dyad members<br>b. Support dyadic communication<br>c. Enhanced positive experiences in caregiving<br>d. Maintenance of positive and meaningful social relationships
**Performing activity:** the observable behaviors in the caregiving situation.	The observable actions involving the caregiving dyad. For the purpose of this study, performing activity refers to Psychosocial interventions: "interpersonal interventions concerned with the provision of information, education, or emotional support together with individual psychological interventions addressing a specific health and social care outcome" (4)<br><br>- promotion of adaption, wellbeing and quality of life (1)<br>- staff awareness training (1)<br>- support groups for both PLWD and caregiver (1, 3)<br>- recognizing care needs (1)<br>- involvement in social activities (1)<br>- activity-based therapies (1)	Interpersonal interventions concerned with the provision of information, education, or emotional support together with individual psychological interventions addressing a specific health and social care outcome	Psychosocial interventions aiming to support:<br><br>a. Shared activities for the caregiving dyad to engage in together/activities for the PLWD facilitated by carer<br>b. Activity-based therapies involving both dyad members<br>c. Carer education/training (in interaction with care recipient)

			d. Support groups for both dyad members/focusing on the caregiving relationship
**Fine tuning:**<br><br>efforts (over time) to accommodate the frailty trajectories and histories in creating enrichment	Accommodation to the dementia disease trajectory and the dyad members personal history in the dyadic activities.<br><br>- (the ability to) preserve autonomy and to solve problems in daily life (1)<br>- adapting and coping with practical and emotional consequences of dementia (1, 3)<br>- promotion of adaption, wellbeing and quality of life (1)<br>- building assets, focusing on potential and overcoming the consequences of the disease on personal wellbeing (1)<br>- supporting the dyad to adapt and cope with changing abilities and limitations (1, 2)<br>-recognizing carer needs (1)	The influence of social and environmental resources in finding a balance between capacities and limitations. This refers to interventions that are intended to support caregivers to adapt an activity or environment, to support the care recipient to participate in valued activities.	a. Building assets, focusing on potential, and overcoming the consequences of the disease on personal wellbeing<br>b. Supporting the dyad members to adapt and cope with changing abilities and limitations<br>c. Strengthen people with dementia in their forces and capabilities

2 References

1. Dröes RM, Chattat R, Diaz A, Gove D, Graff M, Murphy K, et al. Social Health and Dementia: A European Consensus on the Operationalization of the Concept and Directions for Research and Practice. Aging Ment Health (2017) 21(1):4-17. doi: 10.1080/13607863.2016.1254596.
2. Hoel V, Ambugo EA, Wolf-Ostermann K. Sustaining Our Relationship: Dyadic Interactions Supported by Technology for People with Dementia and Their Informal Caregivers. Int J Environ Res Public Health ((In press)) 19.
3. Hoel V, Wolf-Ostermann K, Ambugo EA. Social Isolation and the Use of Technology in Caregiving Dyads Living with Dementia During Covid-19 Restrictions. Frontiers in Public Health (2022) 10. doi: 10.3389/fpubh.2022.697496.
4. Pusey H, Richards D. A Systematic Review of the Effectiveness of Psychosocial Interventions for Carers of People with Dementia. Aging Ment Health (2001) 5(2):107-19. doi: 10.1080/13607860120038302.
