# Supplementary material for: Enrichment of dementia caregiving relationships through psychosocial interventions: A scoping review
Source: Front Med (Lausanne). 2023 Jan 5;9:1069846. doi: 10.3389/fmed.2022.1069846 (PMC9849912; doi:10.3389/fmed.2022.1069846)
Supplement: Supplementary file 3 [file Data_Sheet_1.pdf]

Supplementary Material

1 Medline Search Strategy

1. (retirement home or old age facilit*).mp. or Long-Term Care/
2. (nursing cent*).mp. or Nursing Homes/
3. (assisted living).mp. or Assisted Living Facilities/
4. (retirement home* or old age home or old age facilit*).mp. or Homes for the Aged/
5. (home based).mp. or Home Care Services/
6. (communit* or neighbourhood* neighborhood* or domestic* or domiciliary or home dwelling) or Home Nursing/
7. 1 or 2 or 3 or 4 or 5 or 6
8. dementia.mp. or Dementia/
9. (together* or close* or connect* or intima* or fun* or entertain* or boredom or convers* or social* or positive experience* or reminiscen* or sociali* or social* connected* or social* activ* or meaningful engag* or meaningful occupa* or meaningful* or enrich* or reward* or purpose* or gratif* or valuabl* or fulfil* or share* or relationship* or reciproc* or pleasur*).mp. or Communication/
10. (intervention* or psychosocial).mp. or Psychosocial Intervention/
11. (trial or experiment* or investigat* or rct).mp. or Clinical Trial/ or Randomized Controlled Trial/
12. (therap* or treatment*).mp. or Therapeutics/
13. (awareness or training or person cent* or relationship cent*).mp. or Nurse-Patient Relations/
14. 10 or 11 or 12 or 13
15. (social interaction or participat* or social* engage* or active engagement).mp. or Social Participation/
16. (tailor* or adapt* or support* or social health or assist* or asset* or potential* or capacity* or capabil* or activity analysis or activity gradation).mp. or Psychosocial Functioning/
17. 15 or 16
18. (spouse* or caregiver* or care giver* or dyad* or couple* or famil* or relative* or carer* or staff or nurse* or healthcare worker* or health care worker* or healthcare professional* or health care professional*).mp or Caregivers/ or Health Personnel/
19. 7 and 8 and 9 and 14 and 17 and 18

Initial search in Medline yielded 2757 results (02.02.22)

https://ovidsp.ovid.com/ovidweb.cgi?T=JS&NEWS=N&PAGE=main&SHAREDSEARCHID=SolzNJJGGHEDwk2FCq2cXCjYFDLXoPHJLu5uhuv3A7Qpu2qSZ8f9w6yOZY0W6Lpu

Search	Query	Results
20	Limit 19 to English language	2757
19	7 and 8 and 9 and 14 and 17 and 18	2900
18	(spouse* or caregiver* or care giver* or dyad* or couple* or famil* or relative* or carer* or staff or nurse* or healthcare worker* or health care worker* or healthcare professional* or health care professional*).mp. or Caregivers/ or Health Personnel/	3314114
17	15 or 16	12490599
16	(tailor* or adapt* or support* or social health or assist* or asset* or potential* or capacity* or capabil* or activity analysis or activity gradation).mp. or Psychosocial Functioning/	12296987
15	(social interaction or participat* or social* engage* or active engagement).mp. or Social Participation/	575975
14	10 or 11 or 12 or 13	12751974
13	(awareness or training or person cent* or relationship cent*).mp. or Nurse-Patient Relations/	608463
12	(therap* or treatment*).mp. or Therapeutics/	8387450
11	(trial or experiment* or investigat* or rct).mp. or Clinical Trial/ or Randomized Controlled Trial/	5903638
10	(intervention* or psychosocial).mp. or Psychosocial Intervention/	1105193
9	(together* or close* or connect* or intima* or relationship or fun* or entertain* or boredom or convers* or social* or positive experience* or reminiscen* or sociali* or social* connected* or social* activ* or meaningful engag* or meaningful occupa* or meaningful* or enrich* or reward* or purpose* or gratif* or valuabl* or fulfil* or share* or relationship* or reciproc* or pleasur*).mp. or Communication/	8930361
8	dementia.mp. or Dementia/	126079
7	1 or 2 or 3 or 4 or 5 or 6	854929
6	(communit* or neighbourhood* or neighborhood* or domestic* or domiciliary or home dwelling).mp. or Home Nursing/ [mp=title, abstract, original title, name of substance word, subject heading word, floating sub-heading word, keyword heading word, organism supplementary concept word, protocol supplementary concept word, rare disease supplementary concept word, unique identifier, synonyms]	767099
5	home based.mp. or Home Care Services/	43214
4	(retirement home* or old age home or old age facilit*).mp. or Homes for the Aged/	14844
3	assisted living.mp. or Assisted Living Facilities/	2833
2	nursing cent*.mp. or Nursing Homes/	37851
1	(retirement home or old age facilit*).mp. or Long-Term Care/	27484
